# Pinworm Infection Misdiagnosed With Primary Eosinophilic Colitis. A Case Series

**DOI:** 10.14309/crj.0000000000001547

**Published:** 2024-10-31

**Authors:** Carlo Maria Rossi, Marco Vincenzo Lenti, Antonio Lo Bello, Stefania Merli, Alessandro Vanoli, Andrea Anderloni, Antonio Di Sabatino

**Affiliations:** 1 Department of Internal Medicine and Medical Therapeutics, University of Pavia, Pavia, Italy; 2 First Department of Internal Medicine, Fondazione IRCCS Policlinico San Matteo, Pavia, Italy; 3 Anatomic Pathology Unit, IRCCS San Matteo, Pavia, Italy; 4 Gastroenterology and Digestive Endoscopy Unit, Fondazione IRCCS Policlinico San Matteo, Pavia, Italy

**Keywords:** eosinophil, gut, parasite, worm

## Abstract

Parasitic infections make it difficult to establish a diagnosis of primary eosinophilic gastrointestinal disorders (EGID), may even co-occur with inflammatory bowel disease and complicate the management of patients treated with immunosuppressants. Yet, pinworm infection is often ignored as a cause of mucosal eosinophilia. We described 3 cases where pinworm infection was initially misdiagnosed as primary EGID. No current guideline is available for EGID in adult patients, while for patients with inflammatory bowel disease, parasitic infection screening is recommended only in some subset of patients. A more comprehensive and precise recommendation regarding the parasitic species to consider and the diagnostic tests to order is currently lacking.

## Introduction

Eosinophilic gastrointestinal disorders (EGIDs) are characterized by an eosinophil-rich infiltration of the gut with organ dysfunction. EGID can be categorized into a primary form, in the absence of known causes, and a secondary form, caused by specific etiologies (eg, infections, drugs, inflammatory bowel disease [IBD], and vasculitides). Parasitic infections are frequently responsible for intestinal wall eosinophilia, hampering to establish a diagnosis of primary EGID, or may even co-occur with IBD, complicating the management of immunosuppressant-treated patients. Yet, the screening for pinworm, that is, *Enterobius vermicularis*, is not included in current diagnostic guidelines in the suspicion of primary EGID or IBD.^1–3^ Here, we report 3 cases of adults referred for the suspicion of primary EGID, who were eventually diagnosed with *E. vermicularis* infection.

## Case Report

**Figure 1.**
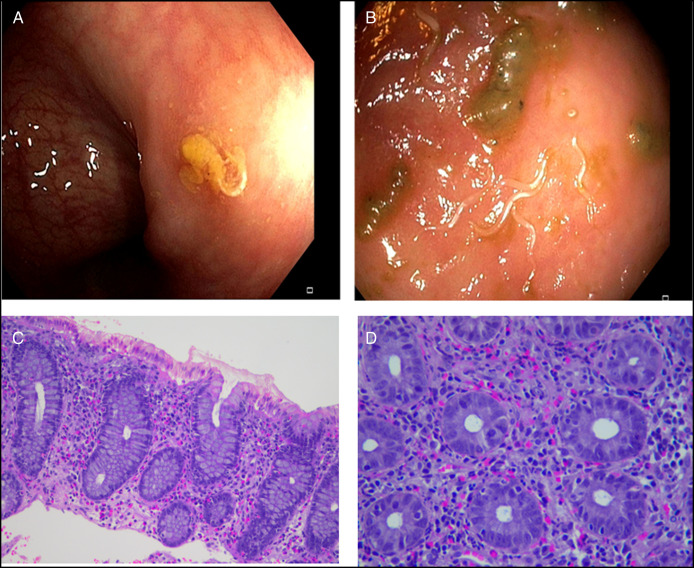
Representative endoscopic images of 2 cases (patient #1, A and patient #2, B). (A) *E. vermicularis* was noted in the sigmoid colon. (B) a marked colonization of *E. vermicularis* is shown in the cecum. (C, 100×) and (D, 200×) representative hematoxylin & eosin staining of histopathological biopsy specimens of the cecum of patient #2 at different magnification. *E. vermicularis*, *Enterobius vermicularis*

Patient #1 was referred with a provisional diagnosis of primary eosinophilic enterocolitis. A colonoscopy was performed due to rectal bleeding and anemia and revealed terminal ileitis with histological findings of active ileocolitis with eosinophilia. Hence, a provisional diagnosis of primary EGID was confirmed. A corticosteroid treatment with enteric-release budesonide was prescribed. Over the following months, the patient reported worsening gastrointestinal symptoms and the occurrence of widespread urticarial and anorectal itch, frequent nocturnal awakenings, and dyspnea. Therefore, 6 months later, a follow-up colonoscopy was performed. Ileum and cecum erosions were found, together with a rich eosinophilic infiltrate at histology (Table 1). Peripheral eosinophilia, an increase of eosinophilic cationic protein and the elevation of fecal calprotectin were found. Of note, a coproparasitological examination on 3 samples and serology for *Strongyloides stercoralis* were negative. Another follow-up colonoscopy was performed one year later and revealed a massive infestation by *E. vermicularis* (Figure 1). Corticosteroids were withdrawn, while mebendazole was prescribed, obtaining symptoms resolution.

**Table 1.** Clinical, laboratory, and histological data of the 3 cases

Parameter	Case #1	Case #2	Case #3
Clinical symptoms			
Weight loss	Absent	Present	Absent
Diarrhea	Absent	Present	Absent
Abdominal pain	Present	Present	Present
GI bleeding	Present	Present	Absent
Pruritus	Present	Absent	Absent
Laboratory test			
Stool ova and parasites	Negative	Negative	Negative
Stool culture	Negative	Negative	Negative
*Helicobacter pylori* fecal antigen test	Negative	Negative	Negative
*Strongyloides* serology	Negative	Negative	Not assessed
Hb (g/dL)	13,3	14	15,5
WBC (n/mcl)	5,230	11,910	7,330
Blood Eo (%)	9.6	4.4	5.8
CRP (mg/dL)	0.02	0.05	0.03
FCAL (µg/g)	63	764	131
ECP (µg/L)	39.6	45.70	/
Scotch test	/	/	Negative
Eosinophilic infiltration (number of Eo/HPF)			
Ileum	>100	0	68
RC	>100	>90	28
TC	>100	>90	20
LC	89	>90	12
Sigma	>100	>90	1
Rectum	47	>90	2

CRP, C-reactive protein; ECP, eosinophil cationic protein; Eo, eosinophil; FCAL, faecal calprotectin; GI, gastrointestinal; Hb, hemoglobin; HPF, high-power field; L, lymphocyte; LC, left colon; RC, right colon; TC, transverse colon; WBC, white blood cells.

Patient #2 was hospitalized for abdominal pain, diarrhea, and vomiting. An abdominal computed tomography scan was performed and revealed a thickening of jejunum with contrast enhancement. Esophagogastroduodenoscopy and colonoscopy were macroscopically normal, but an abundant eosinophilic infiltrate was found (Table 1, Figure 1), including the stomach, the duodenum, the ileum, and the colon, on histological examination. Coproparasitological examination on 3 samples and serology for *S. stercoralis* were negative. Peripheral eosinophilia, increase in serum eosinophilic cationic protein, and fecal calprotectin were observed. A diagnosis of primary eosinophilic gastroenterocolitis was formulated, and a treatment with enteric-release budesonide was started. After improvement, his condition worsened due to severe abdominal pain, unresponsive to opioids, and weight loss. Another colonoscopy was performed a few months later, and *E. vermicularis* was detected.

Patient #3 was referred for abdominal pain and fecal occult blood positivity with a suspicion of ileitis. A colonoscopy was performed, and ileal erosions and edematous inflammation of the ileocecal valve were detected. Histology showed the presence of vasculitis, glandular hypotrophy, and moderate eosinophilic infiltration, but the findings were neither completely diagnostic for IBD nor for EGID (Table 1). A provisional diagnosis of undetermined ileocolitis with eosinophilic infiltration was made. Coproparasitological examination on 3 samples, the scotch-tape test, and stool culture were negative. Another colonoscopy was performed a few months later for persistent symptoms and increased fecal calprotectin (Table 1). At endoscopy, pinworms were detected in the sigmoid colon and after a treatment with ivermectin, clinical improvement was observed.

## Discussion

*E. vermicularis*, also known as pinworm, is a common pathogenic parasite in the world. It affects approximately 4%–38% of the pediatric population, but it also may target adult patients. However, epidemiological data in this setting are scanty, and hence, its relevance is probably underestimated. In Italy, its prevalence is estimated at 13.4%.^4^

Humans are infected by ingesting egg-contaminated water, food, or dust, or indirectly thorough contact with infected people/objects.^1,2^ If symptoms are present, they include perianal pruritus but also insomnia/restlessness and feeding difficulties in children. Seldom, gut infestation may occur, with watery diarrhea and abdominal pain, while extraintestinal manifestations, such as genital-urinary infections, are rare.^3,5,6^

Diagnostically, the stool ova and parasite test is not reliable, since ova and parasites are not passed in the stool, while the scotch-tape test, comprising a night-time application of cellophane tape in the perianal area, has a fundamental role, to detect the adult worms or their eggs.^5^ Pinworms cannot usually penetrate the mucosal barrier without a preexisting lesion; however, some cases of colonic ulceration associated with the infection, mimicking IBD or EGID, are described.^7^ Hence, if the parasite is not detected, a misdiagnosis of primary EGID or IBD may follow.^8,9^

Current guidelines in patients with IBD and pediatric patients with EGID address the issue of parasitic infections. More precisely, guidelines on managing the infections in IBD recommend screening for parasitic infections only in residents of endemic areas.^2^ Guidelines for pediatric EGID conditionally recommend excluding a parasitic infection during the initial evaluation through a microscopic examination of stool for ova and parasites; according to local epidemiology, other additional screening tests may be warranted, such as serologic testing for *Strongyloides* and *Toxocara* species.^3^ The parasites mentioned as a cause of mucosal eosinophilia are mainly tapeworms and hookworms. However, no mention is made to pinworms.

No current guideline is available for EGID in adults. So, while excluding a parasitic infection is indeed essential in the differential diagnosis of mucosal eosinophilia to formulate a diagnosis of EGID, a more comprehensive recommendation regarding the parasitic species to consider is currently lacking. Accordingly, the accuracy of the scotch test varies; if a test performed on a single day has a sensitivity of around 50%, this increases to 90% when performing the test on more days.

Based on the presented cases, a pinworm infection should always be ruled out for both diagnostic and therapeutic purposes in all patients with suspected EGID, through a night-time application of cellophane tape in the perianal area, ideally for 3–5 consecutive days.^7,10,11^ Moreover, this screening should again be performed, especially after starting a corticosteroid therapy. Future guidelines should better address this aspect. Moreover, studies are needed to demonstrate whether a pre-emptive strategy with antiparasitic agents may be warranted in some clinical settings, paralleling the indications for the management of hypereosinophilic syndromes.^12,13^

## DISCLOSURES

Author contributions: MV Lenti, CM Rossi designed the study; A. Lo Bello, CM Rossi, MV Lenti drafted the manuscript and interpreted data with A. Di Sabatino; S. Merli and A. Anderloni enrolled patients, followed them up; A. Vanoli analyzed the patients' samples. AD Sabatino revised the manuscript for important scientific content. All authors revised and approved the final version of the manuscript. A. Di Sabatino is the article guarantor.

Financial disclosure: None to report.

Informed consent was obtained for this case report.

We thank Fondazione IRCCS Policlinico San Matteo for supporting our work.
